# Chromosomal Rearrangements and Origin of the Multiple XX/XY_1_Y_2_ Sex Chromosome System in *Harttia* Species (Siluriformes: Loricariidae) 

**DOI:** 10.3389/fgene.2022.877522

**Published:** 2022-03-21

**Authors:** Geize Aparecida Deon, Larissa Glugoski, Francisco de Menezes Cavalcante Sassi, Terumi Hatanaka, Viviane Nogaroto, Luiz Antônio Carlos Bertollo, Thomas Liehr, Ahmed Al-Rikabi, Orlando Moreira-Filho, Marcelo de Bello Cioffi, Marcelo Ricardo Vicari

**Affiliations:** ^1^ Departamento de Genética e Evolução, Universidade Federal de São Carlos, São Paulo, Brazil; ^2^ Departamento de Biologia Estrutural, Molecular e Genética, Universidade Estadual de Ponta Grossa, Paraná, Brazil; ^3^ Institute of Human Genetics, University Hospital Jena, Jena, Germany

**Keywords:** fish, karyotype evolution, molecular cytogenetics, unstable genomic sites, whole chromosome painting

## Abstract

The Neotropical genus *Harttia* comprises species with extensive chromosomal remodeling and distinct sex chromosome systems (SCSs). So far, three different SCSs with male heterogamety have been characterized in the group. In some species, the presence of the XX/XY_1_Y_2_ SCS is associated with a decrease in diploid numbers and several chromosomal rearrangements, although a direct relation to sex chromosome differentiation has not been shown yet. Here, we aimed to investigate the differentiation processes that have led to the establishment of the rare XX/XY_1_Y_2_ SCS and track its evolutionary history among other *Harttia* species. For that, four whole chromosome painting probes derived from chromosome 1 of *H. torrenticola* (HTO-1), chromosomes 9 and X of *H. carvalhoi* (HCA-9 and HCA-X), and chromosome X from *H. intermontana* (HIN-X) were applied in nine *Harttia* species. Homeologous chromosome blocks were located in *Harttia* species and demonstrated that Robertsonian (Rb) fusions originated HTO-1, HCA-9, and HCA-X chromosomes, while Rb fissions explain Y_1_ and Y_2_ sex chromosomes. Specifically, in *H. intermontana*, HCA-X, HCA-9, and the NOR-bearing chromosome demonstrated that homeologous blocks were used in the HIN-X and metacentric pair 2 origins. Consequently, diploid numbers changed between the studied species. Overall, the data also reinforce the existence of unstable genomic sites promoting chromosomal differentiation and remodeling within the genus *Harttia*.

## Introduction

Although sex determination can be environmentally determined in some species, it is usually genetically regulated, and often associated with the presence of sex chromosomes (Furman et al., 2020). According to a widely accepted model, sex chromosomes arise from an autosomal pair due to the emergence of a sex-specific locus in one of the homologous (Bull, 1983; Charl-esworth, 2002). Over time, the ancestral homologous pair undergoes divergences in its genetic composition, recombination rate, and morphology, leading to sex chromosomes differentiation (Charlesworth et al., 2005; Bachtrog et al., 2014). Thus, sex chromosomes can be recognized according to their size and shape in a karyotype (Ghigliotti et al., 2016). However, sometimes sex chromosomes are indistinguishable concerning their gross morphology, and so defined as homomorphic ones (Chalopin et al., 2015). The most common examples of heteromorphic systems are the XX/XY, where the Y chromosome is restricted to males, and the ZZ/ZW, where the W chromosome is restricted to females (Graves, 2006). Being observed in most mammals and birds, respectively, both systems show different levels of genetic divergence (Graves, 2006; Chalopin et al., 2015). Although little is known about why and how different SCSs have evolved (Tree of Sex Consortium, 2014), the processes associated with their evolutionary origin and differentiation among vertebrates have awoken considerable interest (Devlin and Nagahama, 2002). In fishes, a high diversity of sex-determining mechanisms and SCSs with independent origins can be found (Devlin and Nagahama, 2002; Sember et al., 2021), thus making comparative evolutionary analyzes somewhat difficult. Fishes often present high plasticity concerning sex chromosomes, including none or only subtle changes between the sex pair to major chromosomal rearrangements and size differences (Devlin and Nagahama, 2002).

Among the multiple SCSs, the following types were already identified in fishes X_1_X_1_X_2_X_2_/X_1_X_2_Y, X_1_X_1_X_2_X_2_/X_1_Y_1_X_2_Y_2_, XX/XY_1_Y_2_, Z_1_Z_1_Z_2_Z_2_/Z_1_Z_2_W_1_W_2_, and ZZ/ZW_1_W_2_ (Kitano and Peichel, 2012). While the X_1_X_1_X_2_X_2_/X_1_X_2_Y system is well-represented among several fish families, the XX/XY_1_Y_2_ system is found only in a few (Kitano and Peichel, 2012; Sember et al., 2021). In contrast to simple SCSs, where repetitive DNAs play an essential role in sex chromosome differentiation (Yano et al., 2014; Schemberger et al., 2019), multiple SCSs appear forced by divergent evolutionary trends. It appears that chromosomal rearrangements are more relevant to the evolutionary process of multiple SCSs than the accumulation of repetitive sequences (Almeida et al., 2015). For this reason, molecular cytogenetic procedures based on fluorescence *in situ* hybridization (FISH), e.g., using whole chromosome painting (WCP) probes, has been successfully applied in different fish groups, providing new insights into the differentiation of sex chromosomes, especially for multiple ones (Cioffi et al., 2011; Blanco et al., 2014; Oliveira et al., 2018; Moraes et al., 2019).

*Harttia* is a Neotropical fish group comprising species with distinct diploid numbers and karyotypic variations emerged by extensive evolutionary conserved chromosomal rearrangements (Blanco et al., 2013, 2014, 2017; Deon et al., 2020; Sassi et al., 2020; Sassi et al., 2021). The chromosomal number ranges from 2n = 52 to 62, including B chromosomes and different SCSs (Blanco et al., 2012, 2017; Deon et al., 2020; Sassi et al., 2020; Sassi et al., 2021). In phylogenetic reconstructions, three distinct clades were proposed for the genus, thus reinforcing the extensive diversification experienced by the lineage; also, it is grouping the species according to their South American distribution: (I) from the Guyana shield rivers; (II) from the northern Brazilian rivers; and (III) from the Brazilian south/southeast rivers (Londoño-Burbano and Reis, 2021). Three SCSs were detected so far: (1) the X_1_X_1_X_2_X_2_/X_1_X_2_Y system, present in *H. punctata*, *H. duriventris,* and *H. villasboas*, and (2) a proto/neo-XX/XY system in *H. rondoni*, both belonging to clade II, and (3) the XX/XY_1_Y_2_ system in *H. carvalhoi, H. intermontana,* and *Harttia* sp.1, species which belong to clade III (Centofante et al., 2006; Blanco et al., 2017; Sassi et al., 2020; Sassi et al., 2021; Deon et al., submitted). Chromosomal data compared to a phylogenetic framework indicate that ancestral karyotype with 2n = 58 chromosomes and without a differentiated SCS could represent a plesiomorphic condition for clade III (Deon et al., submitted). Belonging to the same clade III, the species *H. torrenticola* has a karyotype composed by 2n = 56, undifferentiated sex chromosomes (Blanco et al., 2013), and a large metacentric pair being morphologically similar to the X chromosome of *H. carvalhoi*. WCP-FISH experiments using X_1_ and X_2_ probes derived from *H. punctata*, confirmed that chromosomes that gave rise to the X_1_X_2_Y and the XY_1_Y_2_ systems are evolutionary independent (Deon et al., submitted).

Here, we aimed to investigate the differentiation processes that have led to the establishment of the rare XX/XY_1_Y_2_ SCS and to track its evolutionary history among other *Harttia* species. For that, we performed a WCP-FISH investigation using four distinct sex chromosome-specific probes hybridized in several species. The results allowed us to identify the main rearrangements involved in the origin of this unique SCS. Besides, the data provide new insights into the origin and evolution of such a rare XY-derived SCS, consequently increasing our knowledge about the evolution of vertebrate sex chromosomes.

## Materials and Methods

### Individuals and Chromosome Preparation

Representatives of *Harttia* species analyzed in this study are summarized in Table 1. Specimens were collected with the authorization of the Chico Mendes Institute for Biodiversity Conservation (ICMBIO), System of Authorization and Information about Biodiversity (SISBIO-Licenses No. 10538-3 and 15117-2), and National System of Genetic Resource Management and Associated Traditional Knowledge (SISGEN-A96FF09), Brazil. Species were identified based on their morphological features by Dr. Oswaldo Oyakawa curator of the fish collection of the Museu de Zoologia da Universidade de São Paulo (MZUSP), Brazil.

**TABLE 1:** Collection sites of the studied species, diploid chromosome number (2n), and sample size (N).

Species	2n	Locality	N
*H. carvalhoi*	♀52, XX	Grande stream, Pindamonhangaba – SP (22°47′8″S 45°27′19″W)	17♀, 12♂
	♂53, XY_1_Y_2_		
*Harttia* sp. 1	♀56, XX	Macacos stream, Silveiras – SP (22°40′43.0″S 44°51′25.0″W)	10♀, 7♂
	♂57, XY_1_Y_2_		
*H. intermontana*	♀52, XX	Piranga river, Carandaí – MG (20°59′34.0″S 43°43′30.0″W)	20♀, 13♂
	♂53, XY_1_Y_2_		
*H. punctata*	♀58, X_1_X_1_X_2_X_2_	Bandeirinha river, Formosa – GO (15°19′25″S 47°25′26″W)	18♀,25♂
	♂57, X_1_X_2_Y		
*H. kronei*	58♀♂	Açungui river, Campo Largo – PR (25°22′44″S 49°39′08″W)	10♀, 5♂
*H. gracilis*	58♀♂	Machadinho stream, Santo Antônio do Pinhal – SP (22°48′31″S 45°41′21″W)	18♀,15♂
*H. longipinna*	58♀♂	São Francisco river, Pirapora – MG (17°21′22.8″S 44°51′0.2″W)	13♀,16♂
*H. loricariformis*	56♀♂	Paraitinga river, Cunha – SP (22°52′22″S 44°51′0.2″W)	7♀, 3♂
*H. torrenticola*	56♀♂	Araras stream, Piumhi – MG (20°16′15″S 45°55′39″W)	8♀, 6♂

SP, São Paulo; MG, Minas Gerais; PR, Paraná; GO, Goiás Brazilian states.

Mitotic chromosomes were obtained from kidney cells, according to Bertollo et al. (2015). All procedures agreed with the Ethics Committee of Animal Usage of the Universidade Federal de São Carlos (Process number CEUA 1853260315), Brazil.

### Chromosome Microdissection, Probe Preparation, and Labeling

**FIGURE 1:**
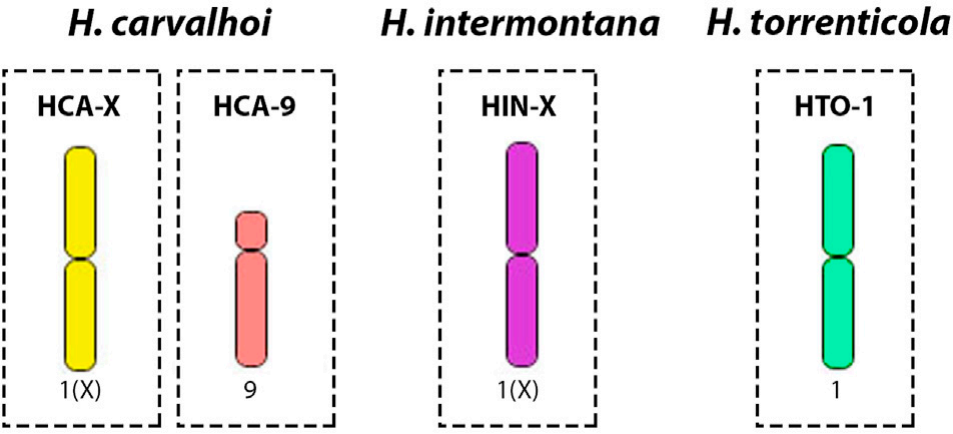
Schematic representation of the microdissected chromosomes used for probe construction in comparative WCP-FISH analyses. 1) the X chromosome of *H. carvalhoi* (HCA-X, in yellow); 2) chromosome 9 of *H. carvalhoi* (HCA-9, in light pink); 3) the X chromosome of *H. intermontana* (HIN-X, in purple); 4) chromosome 1 of *H. torrenticola* (HTO-1, in green).

Fifteen copies of each target chromosome were isolated by glass-needle-based microdissection, and obtained DNA was amplified by oligonucleotide primed-polymerase chain reaction (DOP-PCR) as described in Yang et al. (2009). Chromosomes were chosen based on their morphology - bi-armed chromosomes that were suspected to be originated from Robertsonian fusions were targeted: the largest metacentric (HCA-X), and the largest submetacentric (HCA-9) from *H. carvalhoi*; the largest metacentric (HIN-X) from *H. intermontana*, and the largest metacentric (HTO-1) from *H. torrenticola* (Figure 1). Probes were labeled with Spectrum Orange-dUTP or Spectrum Green-dUTP (Vysis, Downers Grove, United States) in a secondary DOP-PCR, using 1 μL of the primarily amplified product as a template DNA (Yang and Graphodatsky, 2009).

### Fluorescence *in situ* Hybridization

Slides were prepared and pre-treated according to Yang et al. (2009) and denatured in 70% formamide/2x SSC for 3 min at 72°C. For each slide, 12 μL of hybridization solution (containing 0.2 μg of each labeled probe, 50% formamide, 2×SSC, 10% dextran sulfate, and 5 μg of salmon sperm DNA) was denatured for 10 min at 75°C and incubated to pre-hybridize for 1 h at 37°C. To block the hybridization of high-copy repeat sequences, 20 μg of C_*0*_t-1 DNA, directly prepared from *H. carvalhoi, H. torrenticola,* and *H. intermontana* male genomes were used, according to Zwick et al. (1997). Hybridization took place for 48 h at 37°C in a moist chamber. Post-hybridization washes were performed in 1×SSC for 5 min at 65°C, and 5 min in 4×SSC/Tween at room temperature. Finally, the slides were counterstained with 4’,6-diamidino-2-phenylindole (DAPI) in Vectashield mounting medium (Vector, Burlingame, CA, United States).

### Image Analyses and Processing

Metaphase plates were captured using an Olympus BX50 light microscope (Olympus Corporation, Ishikawa, Japan) coupled with a CoolSNAP camera. The images were processed using Image-Pro Plus 4.1 software (Media Cybernetics, Silver Spring, MD, United States). The figures were edited and organized using Adobe Photoshop CC 2020 (San Jose, CA, United States) software.

## Results

**FIGURE 2:**
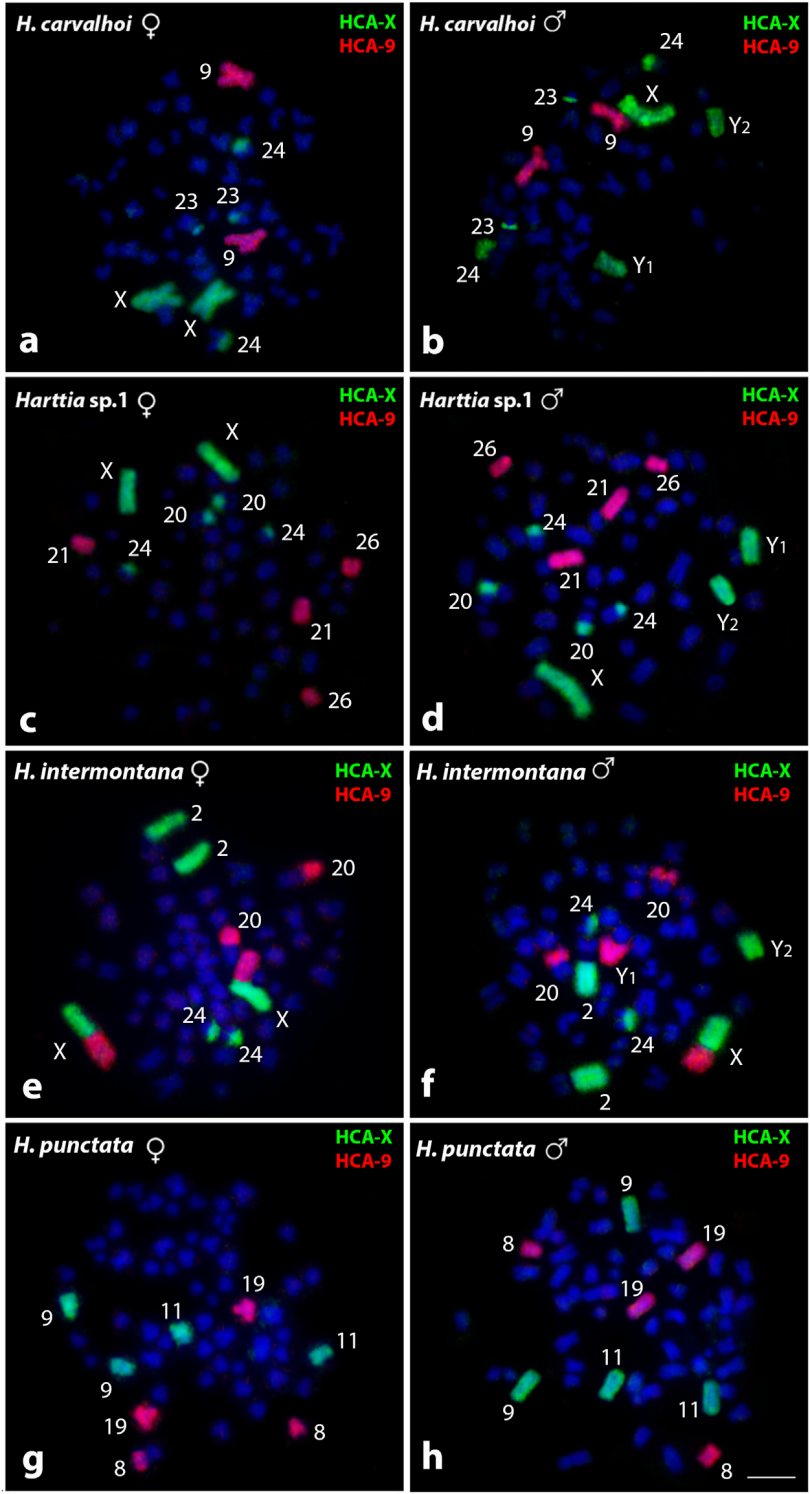
Whole chromosome painting by FISH using HCA-X (green) and HCA-9 (red) probes among *Harttia* species that possess SCSs. The numbers of the labeled chromosome pairs are highlighted in the images. In **(A, B)** metaphases of *H. carvalhoi* female and male, respectively; **(C, D)** metaphases of *Harttia* sp. 1 female and male, respectively; **(E, F)** metaphases of *H. intermontana* female and male, respectively; and **(G, H)** metaphases of *H. punctata* female and male, respectively.Bar = 5 µm.

Results obtained by HCA-X and HCA-9 probes are summarized in Figure 2 and Table 2. In *H. carvalhoi* (52♀/53♂ - XX/XY_1_Y_2_), the HCA-X probe successfully identified their X chromosomes in females and the X, Y_1,_ and Y_2_ chromosomes in males. Small centromeric signals in both acrocentric pairs 23 and 24 were also evidenced. In agreement, the HCA-9 probe correctly recognized the submetacentric pair 9 (Figures 2A,B). Similarly, in *Harttia* sp. 1 (56♀/57♂ - XX/XY_1_Y_2_), the HCA-X probe detected the X chromosome pair in females and the X, Y_1_, and Y_2_ chromosomes in males, besides small centromeric signals in both 20 and 24 acrocentric pairs (Figures 2C,D). The HCA-9 hybridized to 21 and 26 acrocentric pairs (Figures 2C,D). In females of *H. intermontana* (52♀/53♂ - XX/XY_1_Y_2_), the HCA-X probe stained the long (q) arms of the chromosomes X and 2 (Figure 2E). In males, this probe gave signals on Xq, the Y_2_ chromosome, and the 2q (Figure 2F), as well as in the centromeric region of the pair 24 in both males and females (Figures 2E,F). The HCA-9 probe detected the short (p) arms of the X chromosome and the 20q distal region in females, and the Xp arms, the Y_1_ chromosome, and the 20q distal region in males (Figures 2E,F). In *H. punctata* (58♀/57♂ - X_1_X_1_X_2_X_2_/X_1_X_2_Y), the HCA-X probe hybridized on the submetacentric pairs 9 and 11, while the HCA-9 probe showed signals on the metacentric pair 8 and subtelocentric pair 19 (Figures 2G,H).

**TABLE 2:** Main localization of the WCP probes in *Harttia* species. Some small signals were not considered. SCSs means sex chromosome systems.

	Species	HCA-Xprobe	HCA-9
XX/XY_1_Y_2_ system	*H. carvalhoi* ♀	X Chr	Chr. 9
	*H. carvalhoi* ♂	X, Y_1_ and Y_2_ Chr	Chr. 9
	*Harttia* sp. 1 ♀	X Chr	Chr. 21 and 26
	*Harttia* sp. 1 ♂	X, Y_1_ and Y_2_ Chr	Chr. 21 and 26
	*H. intermontana* ♀	Xq and 2q	Xp and 20q distal
	*H.intermontana* ♂	Xq, Y_2_ and 2q	Xp, Y_1_ and 20q distal
X_1_X_1_X_2_X_2_/X_1_X_2_Y system	*H. punctata* ♀♂	Chr. 8 and 19	Chr. 9 and 11
Without differentiated *SCS*s	*H. kronei*	Chr. 17 and 19	Chr. 8 and 13
	*H. gracilis*	Chr. 11 and 22	Chr. 10 and 21
	*H. longipinna*	Chr. 15 and 17	Chr. 7 and 10
	*H. loricariformis*	Chr. 9 and 20	Chr. 8 and 11
	*H. torrenticola*	Chr. 1	Chr. 8 and 23

p, short arms; q, long arms; Chr., chromosome.

**FIGURE 3:**
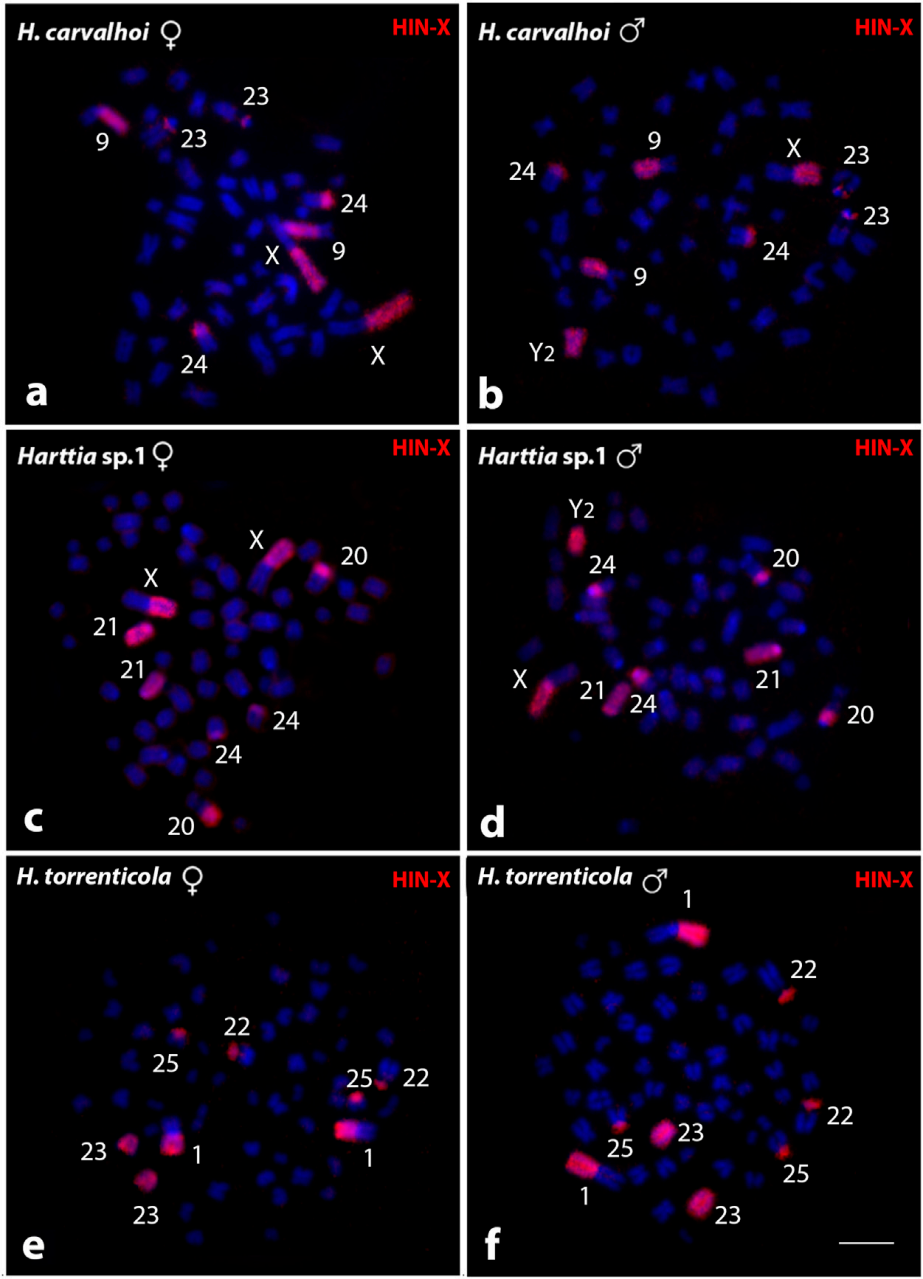
Whole chromosome painting by FISH using the HIN-X probe (red) among *Harttia* species that shared a large metacentric pair. The numbers of the labeled chromosome pairs are highlighted in the images. In **(A, B)** metaphases of *H. carvalhoi* female and male, respectively; **(C, D)** metaphases of *Harttia* sp. 1 female and male, respectively; and **(E, F)** metaphases of *H. torrenticola* female and male, respectively. Bar = 5 µm.

The HTO-1 probe, derived from *H. torrenticola*, showed the same results obtained with the HCA-X probe when tested on those species with an identical large metacentric pair (Supplementary Figure S1). In contrast, the HIN-X probe, from *H. intermontana*, showed different results than those obtained applying HCA-X and HTO-1 probes (Figure 3). In *H. carvalhoi*, HIN-X hybridized on the Xq and 9q arms in females (Figure 3A), and on the Xq arms, Y_2_ chromosome, and 9q arms in males (Figure 3B). In *Harttia* sp. 1, HIN-X labeled the Xq arms and the acrocentric 21 pair in females (Figure 3C), and these same chromosomes, as well as the Y_2_ chromosome, in males (Figure 3D). In *H. torrenticola*, HIN-X stained the 1q arms and the acrocentric 23 pair (Figures 3E,F).

**FIGURE 4:**
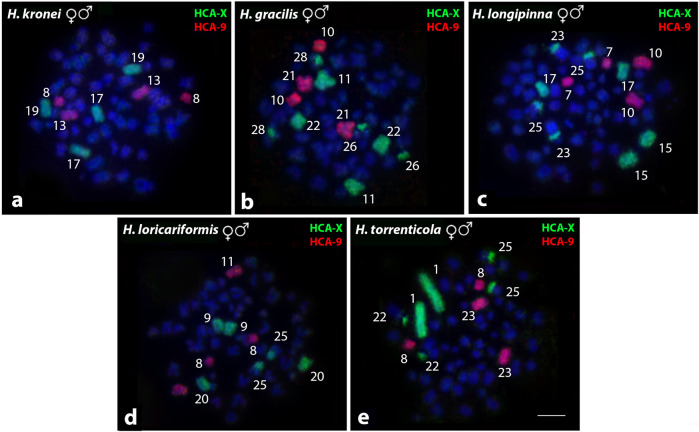
Whole chromosome paint by FISH using HCA-X (green) and HCA-9 probes (red) in *Harttia* species without heteromorphic SCS. The numbers of the labeled chromosome pairs are highlighted in the images. Metaphases of *H. kronei* **(A)**, *H. gracilis* **(B)**, *H. longipinna* **(C)**, *H. loricariformis* **(D)**, and *H. torrenticola* **(E)**.Bar = 5 µm.

*Harttia* species without the heteromorphic sex chromosomes were also used as targets for the comparative WCP-FISH using the HCA-X and HCA-9 probes (Figure 4; Table 2). In *H. kronei* (58♀♂) HCA-X hybridized in the subtelocentric pairs 17 and 19, while the HCA-9 hybridized in chromosome pairs 8 and 13 (Figure 4A). In *H. gracilis* (58♀♂), HCA-X marked the submetacentric 11 and the subtelocentric 22, besides centromeric signals in the acrocentric pairs 26 and 28 (Figure 4B), and the HCA-9 probe was detected in chromosomes 10 and 21 (Figure 4B). In *H. longipinna* (58♀♂), HCA-X hybridized in the subtelocentric pairs 15 and 17, besides the centromeric region of the acrocentric pairs 23 and 25, and HCA-9 hybridized in the metacentric 7, and submetacentric 10 (Figure 4C). In *H. loricariformis* (56♀♂), HCA-X was detected in the submetacentric pair 9, subtelocentric 20, and the centromeric region of the chromosome 25 (Figure 4D), while the HCA-9 probe hybridized in chromosome pairs 8 and 11 (Figure 4D). Finally, in *H. torrenticola* (56♀♂), HCA-X hybridized in chromosome 1 and the centromeric region of the acrocentric pairs 22 and 25, while the HCA-9 probe presents signals of hybridization in pairs 8 and 23 (Figure 4E).

## Discussion

**FIGURE 5:**
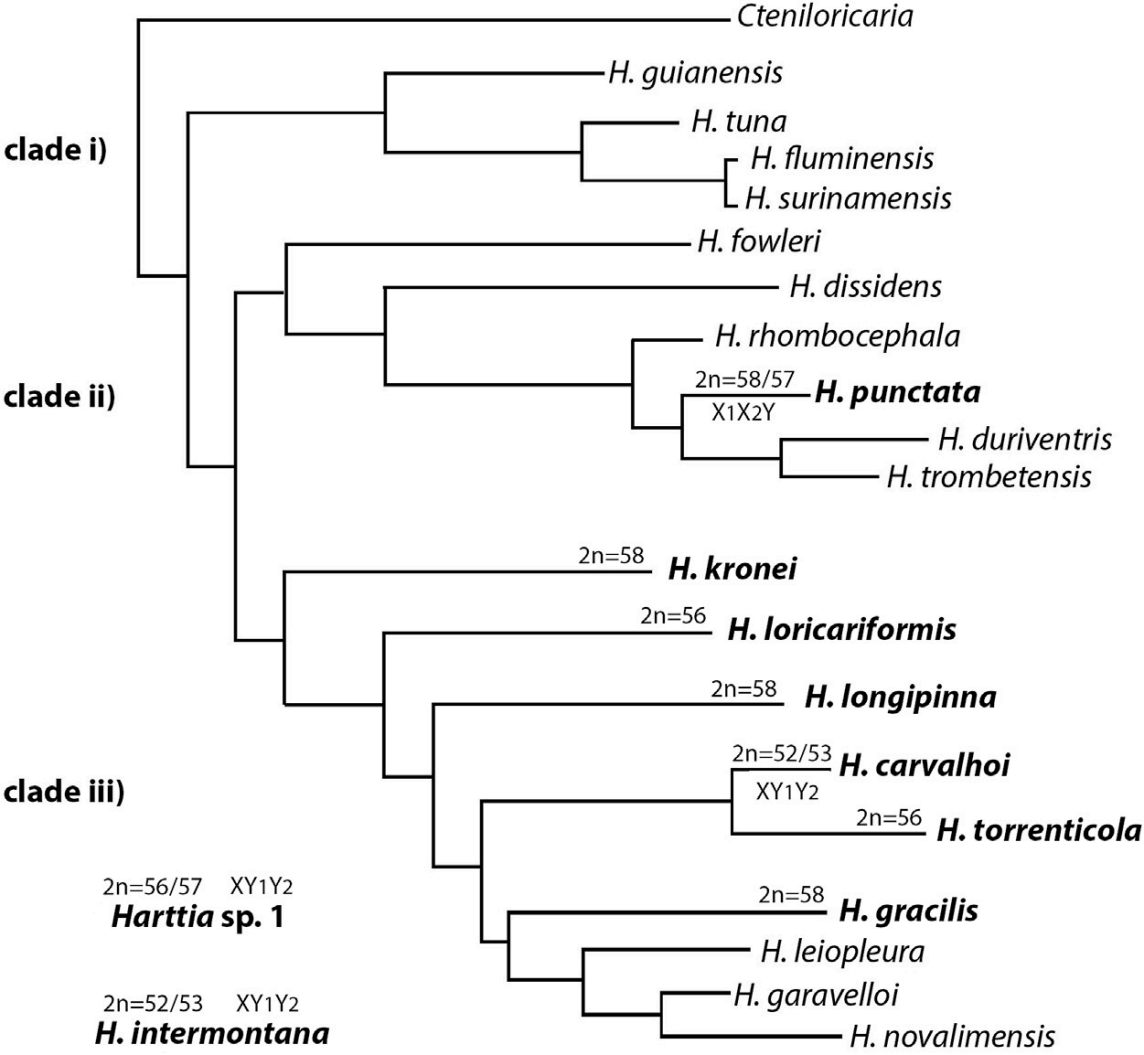
Schematic representation of the phylogenetic relationships among *Harttia* species adapted from the Londoño-Burbano and Reis (2021). *Harttia* sp.1 and *H. intermontana* are not being represented since they were not included in such previous analysis.

**FIGURE 6:**
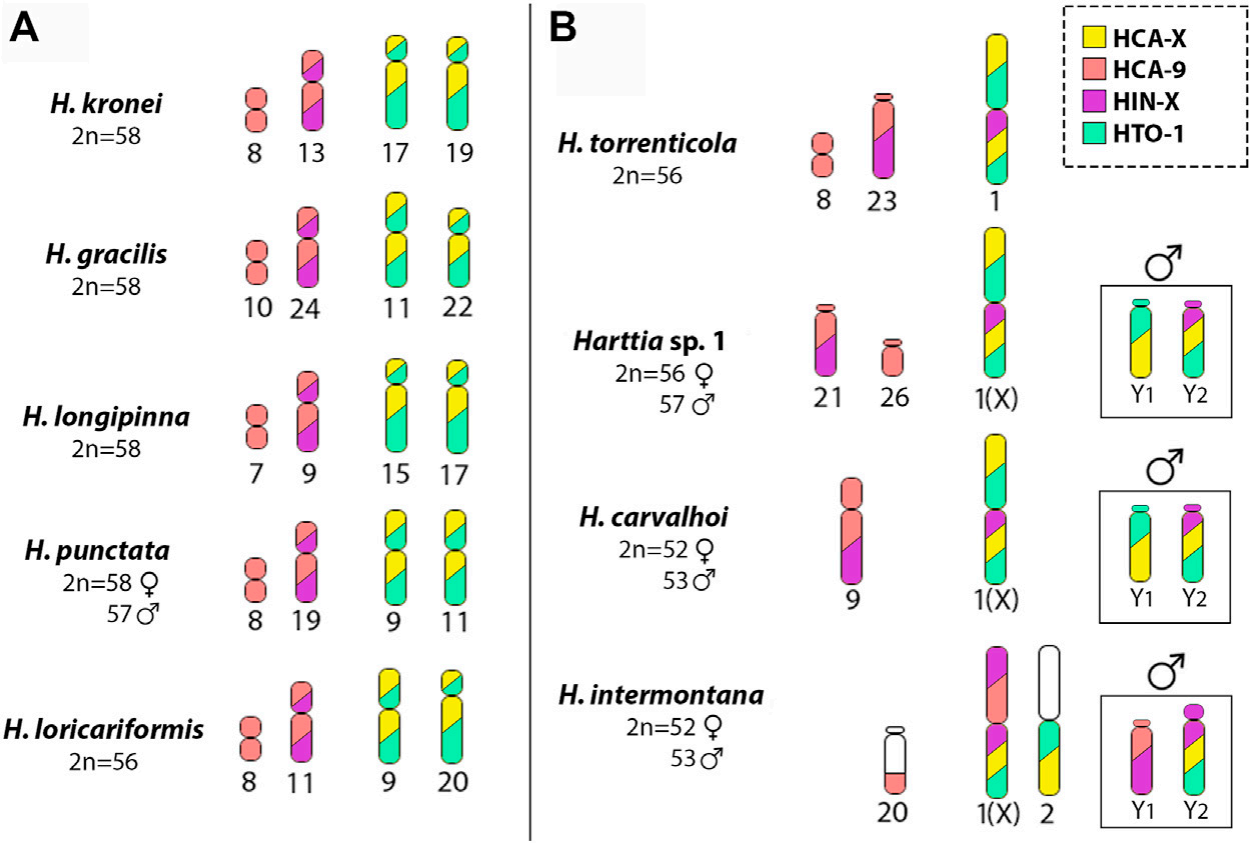
Schematic representation summarizing the distribution of the WCP probes obtained in this study: HCA-X (yellow), HCA-9 (light pink), HIN-X (purple) and HTO-1 (green) in *Harttia* species **(A)** without the largest metacentric chromosome pair -*H. kronei, H. gracilis, H. longipinna, H. loricariformis,* and *H. punctata*- and **(B)** with the largest metacentric pair in the karyotype -*H. carvalhoi, H. torrenticola, H. intermontana* and *Harttia* sp. 1. The highlighted boxes show the male condition and the different composition of the Y_1_ and Y_2_ chromosomes in *H. carvalhoi, H. intermontana* and *Harttia* sp. 1. Note the overlapping of the HCA-9 and HIN-X probes (light pink and purple), and the HCA-X and HTO-1 (yellow and green) probes.

In *Harttia* species, diploid numbers range from 52 to 62 chromosomes (Deon et al., 2020; Sassi et al., 2020; Sassi et al., 2021). Data from phylogeny reconstructions indicate that 58 chromosomes and no large biarmed chromosomes could correspond to a plesiomorphic karyotype condition for species distributed on south and southeast Brazilian drainages—the clade III (Deon et al., submitted). These chromosomal features (Figure 5) include the absence of morphologically differentiated sex chromosomes and a single location of the 5S and 45S rDNA sites in medium-sized bi-armed chromosomes (Deon et al., 2020; Deon et al., submitted). Here, the ancestral reconstructions of the *Harttia* karyotype, using both HCA-X and HCA-9 probes, demonstrated that two chromosome pairs were probably related to the origin of the *H. carvalhoi* chromosomes X and 9 (Figure 6). Thus, *in situ* localizations also reaffirm the role of Robertsonian fusions as the main rearrangements responsible for reducing the diploid number in *H. carvalhoi*. These homeologous chromosome pairs (unfused chromosomes) are shared by *H. kronei*, *H. loricariformis*, *H. longipinna,* and *H. gracilis* (Figure 6). As common features, *H. kronei*, *H. longipinna,* and *H. gracilis* kept 2n = 58 chromosomes and the absence of morphologically differentiated SCSs (Blanco et al., 2017), with chromosomal diversification events mainly occurring by repositioning of the rDNA sites in their karyotypes (Deon et al., 2020; Deon et al., submitted). Although *H. loricariformis* decreased the diploid number to 2n = 56, this species shares the homologous chromosome pairs to HCA-X and HCA-9 as highlighted in *H. kronei* by WCP-FISH. The presence of interstitial telomeric sites in a subtelocentric chromosome of *H. loricariformis* karyotype suggests an origin by Robertsonian fusions (Blanco et al., 2017). The current data thus support the hypothesis on the occurrence of a chromosomal fusion event in *H. loricariformis* karyotype, and that this corresponds to an independent evolutionary event being not associated with the chromosomes X and 9 of *H. carvalhoi*.

Data also showed that the chromosomal rearrangements that led to the XX/XY_1_Y_2_ SCS were triggered within the branch with *H. torrenticola* (Figure 5). The phylogenetic branch grouping *H. carvalhoi* and *H. torrenticola* (Covain et al., 2016; Londoño-Burbano and Reis, 2021; Figure 5) was diversified by Robertsonian fusions, initially giving rise to a large metacentric pair, like that found in the *H. torrenticola* karyotype. Indeed, the large homeologous chromosome regions shared between the chromosomes 1 of *H. torrenticola* (HTO-1) and the X chromosome of *H. carvalhoi* and *Harttia* sp. 1, corroborate that a single evolutionary event of chromosomal fusion would have generated the large metacentric pair in these species. Although *H. torrenticola* does not show sex chromosome heteromorphism related to the metacentric chromosome 1, in *H. carvalhoi* and *Harttia* sp. 1, this chromosome corresponds to the X sex chromosome, with additional rearrangements triggering the origin of the Y_1_ and Y_2_ chromosomes. According to former suggestions (Blanco et al., 2017; Deon et al., 2020) centric fission on the largest metacentric formed the Y_1_ and Y_2_ chromosomes in *Harttia* sp. 1, which are also shared by *H. carvalhoi*. The Y_1_ and Y_2_ positive hybridizations using the HCA-X probe reiterate that centric fission is the main rearrangement related to the origin of the multiple XX/XY_1_Y_2_ SCS of *H. carvalhoi* and *Harttia* sp. 1.

**FIGURE 7:**
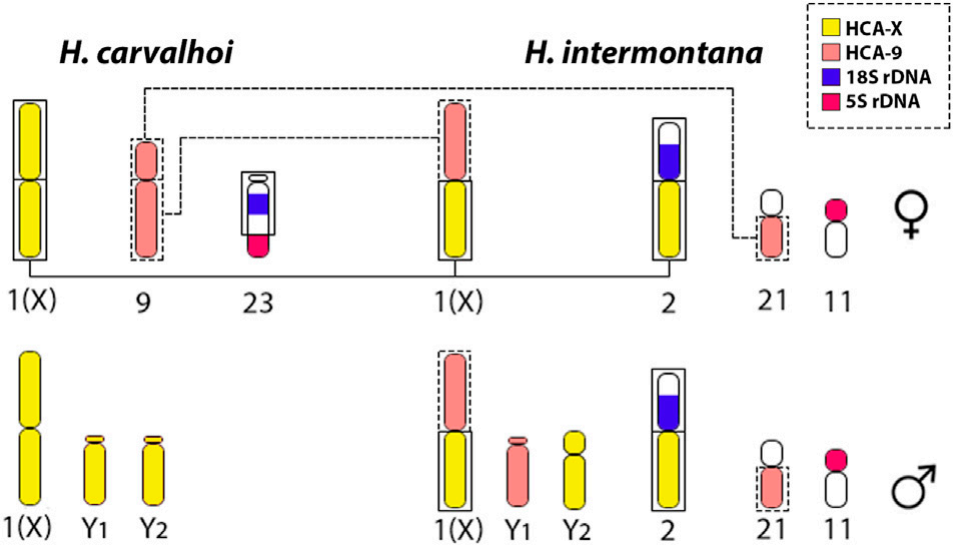
Schematic model representing the rearrangements occurred from *H. carvalhoi* to *H. intermontana*, and the evolvement of HCA-X (yellow) and HCA-9 probes (light pink) on the origin of the XX/XY_1_Y_2_ SCS. Considering *H. intermontana,* a derived species from *H. carvalhoi*, a centric fusion between the long arms of the chromosome 1 (X) and the long arms of the chromosome 9 of *H. carvalhoi*, lead the origin of the X chromosome in *H. intermontana*. An additional fusion with part of the X chromosome and part of the chromosome 23 (bearing the 18S rDNA site) gave rise to the second-largest metacentric chromosome pair in *H. intermontana* (pair 2). It is worth mentioning that the different origin of the X chromosome directly reflects on the genomic composition of the Y chromosomes: while the Y_1_ chromosome corresponds to the long arms of the chromosome 9, the Y2 chromosome corresponds to a part of the X chromosome of *H. carvalhoi.*

However, different from *Harttia* sp. 1 (2n = 56 in females and 57 in males), *H. carvalhoi* diversified its karyotype by other chromosomal fusions, reducing the diploid number to 2n = 52 in females and 2n = 53 in males. Based on the HCA-9 WCP-FISH experiments a chromosome fusion between the subtelocentric pairs 21 and 26, like those found in *Harttia* sp*.* 1, triggered the origin of pair 9 of *H. carvalhoi* (Figures 5, 6). In fact, despite some morphological alterations, this chromosome pair is represented by two other, homologous pairs in *H. kronei*, *H. loricariformis*, *H. longipinna*, *H. gracilis*, and *H. torrenticola*. Based on conventional cytogenetic studies, Deon et al. (2020) proposed the same origin of the XX/XY_1_Y_2_ system in *H. carvalhoi*, *Harttia* sp. 1, and *H. intermontana*. However, the use of the HCA-X, HCA-9, and HIN-X probes enabled now to evidence that additional rearrangements are associated with the XX/XY_1_Y_2_ system of *H. intermontana*. The X chromosome of this species comprises the 9q arms and one arm of the X chromosome of *H. carvalhoi*, indicating a reciprocal translocation between these two chromosome pairs in its origin (Figure 6). After that, centric fission in one of the X chromosomes, followed by a pericentric inversion in one of the resulted elements, generated the Y_1_ and Y_2_ chromosomes in males (Figure 7). It is relevant to notice that the Y_1_ chromosome of *H. intermontana* is derived from chromosome 9 of *H. carvalhoi*, thus different from the Y_1_ chromosome of *H. carvalhoi* and *Harttia* sp. 1. In the same way, the metacentric pair 2 of *H. intermontana* was originated from species-specific chromosomal rearrangements, implying a translocation between the acrocentric chromosome bearing the 45S site and one X chromosome arm. Indeed, the chromosome pair of *H. intermontana* bears the 45S rDNA locus (Deon et al., 2020), a site prone to breaks in *Harttia* karyotypes, leading to extensive chromosomal remodeling events (Deon et al., 2020; Deon et al., submitted).

According to molecular-phylogenetic reconstructions, *H. punctata* - 2n = 58♀/57♂, X_1_X_1_/X_2_X_2_/X_1_X_2_Y (Blanco et al., 2014) - belongs to *Harttia*’s clade II (Covain et al., 2016; Londoño-Burbano and Reis, 2021), and the WCP results here obtained evidenced a similar hybridization condition to those found in *H. kronei*, i.e., the HTO-1, HCA-X, HCA-9, and HIN-X chromosomes were not related to the karyotype diversification of *H. punctata*, highlighting a probable plesiomorphic condition.

It was demonstrated that sex chromosomes could emerge independently and follow distinct differentiation patterns, even among closely related species (Cioffi et al., 2013). Our WCP-FISH data also indicated independent origins for the X_1_X_2_Y and XY_1_Y_2_ SCSs of *Harttia* lineage, as previously proposed (Deon et al., 2020; Sassi et al., 2020). The X chromosome of the XX/XY_1_Y_2_ system originated by fusion of two autosome pairs, leading to the largest metacentric in the karyotype. This fusion could set up a putative homomorphic XX/XY SCS, with subsequent centric fission originating the Y_1_ and Y_2_ chromosomes, as proposed by Blanco et al. (2013). Thus, a set of diverse chromosomal rearrangements probably triggered the differentiation of the same or different SCSs within the *Harttia* lineage, suggesting that sex chromosome turnover may play an important role in the speciation processes of this group.

Evolutionarily conserved breakpoint regions (ECBRs), inside or adjacent to rDNA clusters, were proposed to occur in some Loricariidae lineages, leading to extensive chromosomal remodeling (Barros et al., 2017; Glugoski et al., 2018; Deon et al., 2020; Deon et al., submitted). In the *Harttia* clade III from the south/southeast Brazilian region, several rearrangements adjacent to the rDNAs sites have been extensively reused in the chromosomal diversification (Deon et al., 2020; Deon et al., submitted), including the origin of the X_1_X_1_X_2_X_2_/X_1_X_2_Y SCS in *Harttia* clade II (Deon et al., submitted). In contrast, as rDNAs were not involved in the origin of the XX/XY_1_Y_2_ system, other unstable sites likely occur in the genomes of the species from *Harttia* clade III, as well.

In addition, some hybridization signals besides those indicating the discussed main rearrangements were also detected in small chromosomal regions. They correspond to a highly rearranged repetitive DNA unit shared among *Harttia* species. In *H. carvalhoi, H. gracilis, H. longipinna, H. torrenticola,* and *Harttia* sp. 1, they localize close to the nucleolar organizer region (NOR) and in a small acrocentric pair. In *H. loricariformis* and *H. intermontana* only the signal close to the NOR site and in the small acrocentric chromosome were detected, respectively. The mechanisms responsible for the instability of *Harttia* genome are not fully understood (Deon et al., 2020; Deon et al., submitted). However, repetitive DNA clusters scattered at some genome locations are likely candidates for chromosomal breaks and rearrangements. Cytogenetic data indicate that these sequences are reused in several chromosome rearrangements, including the Robertsonian ones responsible for the origin of the SCSs and a 2n decrease in *Harttia*.

Interstitial telomeric sites (ITS) are common features in some *Harttia* genomes (Blanco et al., 2017; Deon et al., 2020). *H. carvalhoi* and *H. torrenticola*, for example, present an ITS in the large metacentric chromosome (Blanco et al., 2017), indicating its origin by Robertsonian fusion. In contrast, this ITS was lost during the chromosomal evolution of *Harttia* sp. 1. It is known that ITS are hotspots for breakages (Slijepcevic et al., 1997) and that telomeric DNA damages can be irreparable, causing persistent DNA-damage-response activation (Fumagalli et al., 2012), or remaining as fragile sites (Sfeir et al., 2009). According to Slijepcevic (2016), both ITS and terminal telomeric sequences are naturally prone to breakage, leading to chromosome plasticity. Therefore, the rearrangements observed in the X and 2 chromosomes of *H. intermontana* may have been triggered by the instability generated by the ITS in the X chromosome of *H. carvalhoi*.

## Conclusion

Data obtained by WCP-FISH allowed to highlight small pieces of the complex chromosomal evolution that has taken place in *Harttia* species, with a particular emphasis on the origin of a rare multiple SCS and diploid number decrease. We demonstrated the existence of unstable genomic sites promoting chromosomal differentiation and remodeling, where homeologous chromosome blocks were identified after WCP experiments. Besides, we highlighted the distinct Robertsonian fusions and fissions that were involved in the origin the sex chromosomes. In this context, the genus *Harttia* has proved to be an excellent model for the study of evolution of sexual chromosome systems among Neotropical fish species. Next steps now will include a fine-scale analysis of the genetic content of the sex chromosomes in this group aiming to discover novel sex-determining genes, which is an inevitable next step towards fully understating this puzzling scenario.

## Data Availability

The original contributions presented in the study are included in the article/Supplementary Material, further inquiries can be directed to the corresponding author.

## Supplementary Material

Supplementary Figure S1 Hybridizations using the HTO-1 probe (red) in species that present a large metacentric chromosome pair in the karyotype: **(A)** *H. carvalhoi* female, **(B)** *H. carvalhoi* male, **(C)** *Harttia* sp.1 female, **(D)** *Harttia* sp. 1 male, **(E)** *H. intermontana* female and **(F)** *H. intermontana* male. Bar = 5 µm.
