# Supplementary figures and images for: Chromosomal Rearrangements and Origin of the Multiple XX/XY1Y2 Sex Chromosome System in Harttia Species (Siluriformes: Loricariidae)
Source: Front Genet. 2022 Mar 21;13:877522. doi: 10.3389/fgene.2022.877522 (PMC8977651; doi:10.3389/fgene.2022.877522)

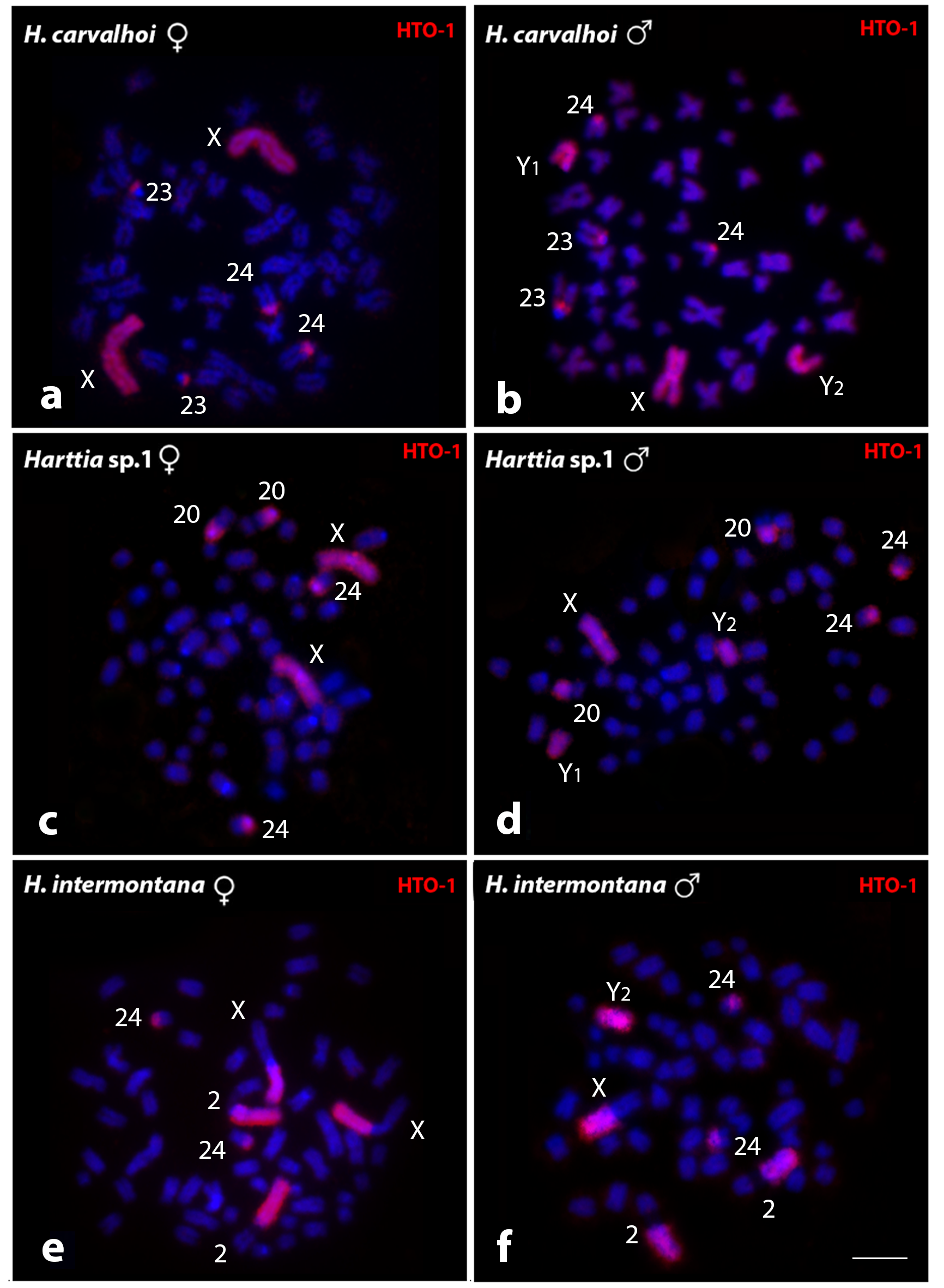

Supplement: Supplementary file 1 [file Image1.TIF]
